# Subcellular trafficking and post-translational modification regulate PIN polarity in plants

**DOI:** 10.3389/fpls.2022.923293

**Published:** 2022-07-27

**Authors:** Shuyang Cheng, Yizhou Wang

**Affiliations:** ^1^Department of Agronomy, College of Agriculture and Biotechnology, Zhejiang University, Hangzhou, China; ^2^Hainan Yazhou Bay Seed Laboratory, Sanya, China; ^3^Zhejiang Provincial Key Laboratory of Crop Germplasm, Zhejiang University, Hangzhou, China

**Keywords:** PINs, auxin transport, polarity, subcellular trafficing, post-translational modification

## Abstract

Auxin regulates plant growth and tropism responses. As a phytohormone, auxin is transported between its synthesis sites and action sites. Most natural auxin moves between cells *via* a polar transport system that is mediated by PIN-FORMED (PIN) auxin exporters. The asymmetrically localized PINs usually determine the directionality of intercellular auxin flow. Different internal cues and external stimuli modulate PIN polar distribution and activity at multiple levels, including transcription, protein stability, subcellular trafficking, and post-translational modification, and thereby regulate auxin-distribution-dependent development. Thus, the different regulation levels of PIN polarity constitute a complex network. For example, the post-translational modification of PINs can affect the subcellular trafficking of PINs. In this review, we focus on subcellular trafficking and post-translational modification of PINs to summarize recent progress in understanding PIN polarity.

## Introduction

Auxin, the first plant hormone to be discovered, participates in many plant developmental processes. Its synthesis, distribution, and degradation respond to a variety of signals, mainly light and gravity; for this reason, auxin function, synthesis, distribution, and degradation have been major focuses of research in plant biology. Auxin is usually synthesized in young cells, such as shoots, leaf primordia, and root tips, and then is redistributed to exert its function. There are two general modes for transporting auxin: long-distance transport and short-distance transport (Michniewicz et al., 2007a). Although auxin can be transported *via* phloem vessels over long distances, it is short-distance transport, which refers to polar transport, that changes the auxin concentration in tissues and enables auxin to affect plant development (Adamowski and Friml, 2015). This polar transport is not powered by gravity, but instead involves active transport between cells. According to the chemiosmotic model, transport over short distances is controlled by auxin transporters (Goldsmith and Goldsmith, 1977). The auxin carriers discovered to date are AUXIN-INSENSITIVE1/LIKE AUX1 (AUX/LAX), NITRATE TRANSPORTER 1.1 (NRT1.1), B SUBFAMILY OF ATP-BINDING CASSETTE (ABCB) family, PIN-FORMED (PIN) family, PIN-LIKE TRANSPORTERS (PILS), and WALLS ARE THIN 1 (WAT1; Zhou and Luo, 2018). Given that the polar localization (asymmetric distribution) of PIN proteins in plants correlates well with the direction of auxin movement, PINs are considered to be the major transporters responsible for asymmetric auxin distributions (Benkova et al., 2003; Swarup and Bennett, 2003; Zhou and Luo, 2018). Noticeably, although the distribution of PINs may determine the auxin flux, the auxin flux or the auxin concentration may influence the polarity of PINs in return (Jonsson et al., 2006; Grigolon et al., 2015).

**Figure 1:**
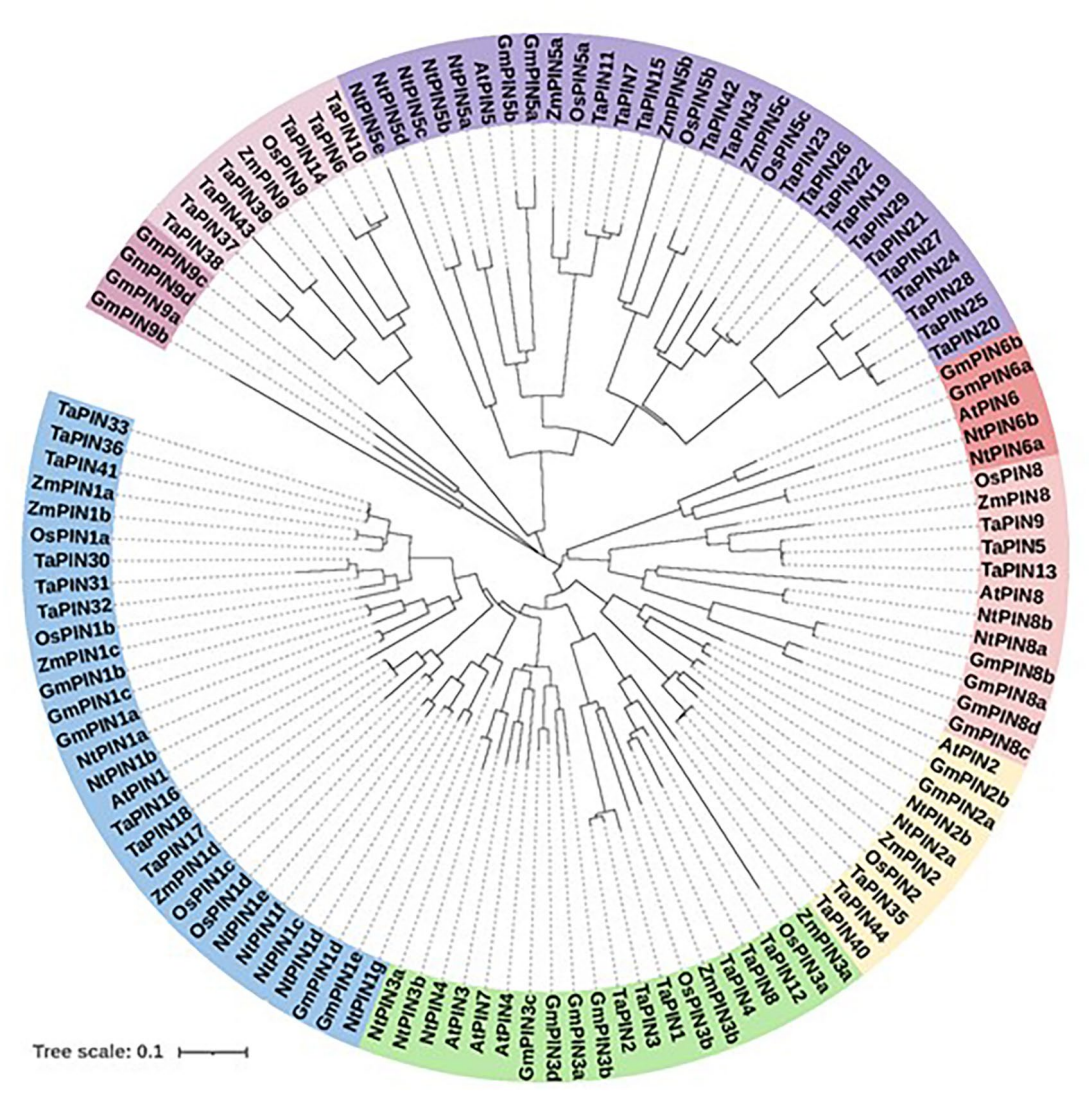
Phylogenetic relationships of the PIN proteins from *Arabidopsis thaliana, Oryza sativa, Zea mays, Glycine max, Nicotiana tabacum*, and *Triticum aestivum*. The protein sequences were downloaded from the NCBI databases, and from recently published data (Wang et al., 2009, 2015; Forestan et al., 2012; Xie et al., 2017; Kumar et al., 2021). The sequences were aligned with ClustalW, and the phylogenetic tree constructed with the neighbor-joining method implemented MEGA version 11 (Tamura et al., 2021).

The PIN family has been identified in at least 30 plant species, and comprises eight genes in *Arabidopsis thaliana* (Zhou and Luo, 2018). In other plant species, most PINs have been studied at the genomic and transcriptional level, and only a small number of PINs have been studied at the gene level (Figure 1; Table 1; Wang et al., 2009, 2015; Miyashita et al., 2010; Forestan et al., 2012; Zhang et al., 2012; Xie et al., 2017; Inahashi et al., 2018; Sun et al., 2018; Li et al., 2019; Gao et al., 2021; Gho et al., 2021; Hou et al., 2021; Kumar et al., 2021; Liu et al., 2022). In *A. thaliana,* PIN proteins are involved in many plant developmental processes and are localized differently in different tissues (Tables 1, 2). During embryogenesis, PIN1, 3, 4, and 7 contribute to the establishment of apical-basal polarity (Friml et al., 2003). These four PINs induce the formation of primordia for aerial and underground organs. Notably, whereas PIN1 plays a major role among these four PINs in aerial organ growth, PIN2 is mainly expressed in roots, where it associates with the aforementioned four PINs to form a local “reflux loop” of auxin to enable the formation of the root meristem (Galweiler et al., 1998; Benkova et al., 2003; Friml et al., 2003; Blilou et al., 2005). In this loop, all PINs can be detected at the basal plasma membrane (PM), but the localization differs in some cells. PIN2 localizes at the apical PM in the root epidermis and lateral root cap, and PIN3 and PIN7 are detectable in the lateral columella (Friml et al., 2003). Given that the role of PIN2 in the “reflux loop” is to transport auxin from the root tip to the root elongation zone and that roots usually bend in the root elongation region, PIN2 is the major carrier involved in root gravitropism (Luschnig et al., 1998; Han et al., 2021). Among PIN3, 4, and 7, which all contribute to auxin lateral flow processes, such as shoot phototropism and gravitropism, as well as lateral root formation, PIN3 is indicated to be the main transporter (Ding et al., 2011; Rakusova et al., 2011; Rosquete et al., 2013). The functions of PIN5, 6, and 8 are less studied (Ding et al., 2012; Lee et al., 2020). In addition, whereas PIN1-4 and PIN7 localize to PM, PIN5, 6, and 8 are in the endoplasmic reticulum (ER), of which PIN6 can be detected at the ER and PM depending on the phosphorylation (Mravec et al., 2009; Ditengou et al., 2018; Sisi and Ruzicka, 2020). The PIN5, 6, and 8 proteins are short PINs, but PM-located PIN proteins are long PINs containing a long hydrophilic loop with some phosphorylation sites that can be phosphorylated by kinases to influence the polarity of the PIN (Bennett et al., 2014; Zhou and Luo, 2018). The polarity of PIN proteins may be influenced by the physical mechanics and can be regulated at least at two levels, namely post-translational modification including phosphorylation, and subcellular trafficking (Heisler et al., 2010; Zhou and Luo, 2018; Ramos et al., 2021). This review mainly focuses on these different regulatory mechanisms.

**Table 1:** Characteristics of PINs in six plant species.

Species	PINs	Highly expressed tissues	Function	References
*Arabidopsis thaliana*	AtPIN1	Embryo, roots meristem and elongation zone, stems, leaves, and flowers	Embryo development, root growth, and flower formation	Galweiler et al., 1998; Friml et al., 2003
	AtPIN2	Roots meristem, elongation and differentiation zone	Root gravitropism	Luschnig et al., 1998; Friml et al., 2003; Blilou et al., 2005
	AtPIN3	Embryo, roots meristem and elongation zone, and stems	Embryo development, root growth, hypocotyl gravitropism and phototropism, and lateral root growth	Luschnig et al., 1998; Friml et al., 2003; Ding et al., 2011; Rakusova et al., 2011; Rosquete et al., 2013
	AtPIN4	Embryo, roots meristem zone, and stems	Embryo development, root growth, hypocotyl gravitropism and phototropism, and lateral root growth	Luschnig et al., 1998; Friml et al., 2003; Ding et al., 2011; Rakusova et al., 2011; Rosquete et al., 2013
	AtPIN5	Hypocotyl, and cotyledon vasculature	Root growth, lateral root growth, and hypocotyl growth	Mravec et al., 2009
	AtPIN6	Shoot apical meristem, hypocotyl, and inflorescence stems	Inhibit inflorescence, and stem elongation	Ditengou et al., 2018
	AtPIN7	Embryo, roots meristem and elongation zone	Embryo development, root growth, hypocotyl gravitropism and phototropism, and lateral root growth	Luschnig et al., 1998; Friml et al., 2003; Ding et al., 2011; Rakusova et al., 2011; Rosquete et al., 2013
	AtPIN8	Roots and pollen	Lateral root growth, and flower growth	Ding et al., 2012; Lee et al., 2020
*Oryza sativa*	OsPIN1a, b, c	Roots, young panicles and base of stems	Root growth, young panicles, and low nitrogen and phosphate response	Sun et al., 2018; Gho et al., 2021; Liu et al., 2022
	OsPIN2	Roots and base of stems	Root growth, lateral root formation and tiller growth	Inahashi et al., 2018
	OsPIN3a, b	Stems, leaves, and young panicles	Root growth	Miyashita et al., 2010; Zhang et al., 2012
	OsPIN5a, b, c	Leaves, shoot apex, and panicles		Wang et al., 2009
	OsPIN8			Wang et al., 2009
	OsPIN9	Base of stems	Tiller growth and ammonium response	Wang et al., 2009; Hou et al., 2021
*Zea mays*	ZmPIN1a, b, c, d	Roots, shoots and endosperm	Root growth and stress response	Forestan et al., 2012; Li et al., 2019
	ZmPIN2	Root tips and male and female inflorescences		Forestan et al., 2012
	ZmPIN5a, b, c	Elongation/mature zone of the primary roots, nodes and young seeds		Forestan et al., 2012
	ZmPIN8	Except roots		Forestan et al., 2012
	ZmPIN9	Roots and nodes		Forestan et al., 2012
	ZmPIN10a, b	Male and female inflorescences		Forestan et al., 2012
*Glycine max*	GmPIN1a, b, c, d, e	Root tips, stems and shoot apical meristems	Root growth and nodule formation	Wang et al., 2015; Gao et al., 2021
	GmPIN2a, b	Roots		Wang et al., 2015
	GmPIN3a, b, c, d	Leaves and flowers		Wang et al., 2015
	GmPIN5a	Leaves, flowers, and nodule		Wang et al., 2015
	GmPIN6a, b	Roots, shoot apical meristems and green pods		Wang et al., 2015
	GmPIN8a b	Leaves and flowers		Wang et al., 2015
	GmPIN9d	Roots, seeds and flowers	Root growth and nodule formation	Wang et al., 2015; Gao et al., 2021
*Nicotiana tabacum*	NtPIN4	Stems and axillary buds	Branching	Xie et al., 2017
*Triticum aestivum*	Unclear	Unclear	Root growth, drought and heat stress response	Kumar et al., 2021

**Table 2:** Polar localization of long PINs in different tissues.

Long-PINs	Shoot primordium		Hypocotyl	Root		References
	Outer cells	Inner future vascular cells		Outer cells	Inner cells	
PIN1	Localize apically toward tips	Localize basally	Localize basally, but change to lateral induced by blue light	Localize basally toward tips		Benkova et al., 2003; Blakeslee et al., 2004; Blilou et al., 2005
PIN2				Localize apically	Localize basally toward tips	Blilou et al., 2005
PIN3			Localize basally, but change to lateral induced by blue light and high ratio of far-red light	Localize laterally	Localize basally toward tips	Friml et al., 2002; Blilou et al., 2005; Keuskamp et al., 2010
PIN4					Localize basally toward tips	Blilou et al., 2005
PIN7				Localize laterally	Localize basally toward tips	Blilou et al., 2005

## Subcellular trafficking of PINs

To date, it has not been determined whether newly synthesized PINs are initially secreted in an apolar or polar manner. Therefore, this review focuses on the post-secretion regulation of PIN. To maintain or change the polarity in developmental processes or after sensing signal changes, such as a change in gravity, PINs can be endocytosed, recycled to the trans-Golgi network (TGN)/early endosome (EE), and then transported to the PM by exocytosis or to the vacuole for degradation by multivesicular bodies (Dhonukshe et al., 2007; Kleine-Vehn et al., 2010; Kitakura et al., 2011; Rodriguez-Furlan et al., 2019). During these processes, many factors affect trafficking by influencing endocytosis, vesicular transport, and membrane fusion, and thus affect PIN polarity (Table 3).

**Table 3:** Factors associated with subcellular-trafficking of PINs.

Factor	Upstream	Function	Study focuses in PINs	Signal	Result	References
CLC2 and CLC3		Endocytosis	PIN3	Blue light	Hook opening and hypocotyl phototropism	Zhang et al., 2017; Hu et al., 2021
				Low red light / far-red light	Hypocotyl elongation	Hu et al., 2021
CPI1		Sterol synthesis	PIN2		Root gravitropism	Men et al., 2008
PIP5K1 and PIPIK2		PI(4,5)P2 synthesis	PIN1 and PIN2		Root gravitropism	Ischebeck et al., 2013
PAX and BRX		Recruit PIP5K	PIN1			Marhava et al., 2020
ROP6/RIC1	LP, PG, TMK1	CME	PIN1 and PIN2		Root gravitropism	Chen et al., 2012; Han et al., 2018; Platre et al., 2019; Pan et al., 2020
14-3-3		Endocytosis	PIN1 and PIN2	Maybe light	Hypocotyl phototropism	Keicher et al., 2017; Reuter et al., 2021
GNOM		Recycling	PIN1			Geldner et al., 2003
GNOM and GNL1		Recycling	PIN2			Teh and Moore, 2007
		Secretory	PIN1			Doyle et al., 2015
SNX1 and VPS29		Recycling	PIN2		Root gravitropism	Jaillais et al., 2006, 2007
CLASP		MT-associated protein and interact with SNX1	PIN2			Ambrose et al., 2013
BEN3/BIG2		Recycling	PIN1		Root gravitropism	Kitakura et al., 2017
BEN1/BIG5	H2O2	Recycling	PIN2	ROS	Stress-induced growth of roots	Zwiewka et al., 2019
ALA3		Interact with GNOM and BIG2	PIN2		Root gravitropism	Zhang et al., 2020
BEX5		Recycling	PIN1			Zhang et al., 2020
RGTB1		Rab-related recycling	PIN1 and PIN3		Communication between the sporophyte and the developing female gametophyte	Rojek et al., 2021a,b
SEC6, SEC8 and EXO70A1		Membrane fusion	PIN1 and PIN2		Root gravitropism	Drdova et al., 2013; Tan et al., 2016
VAMP714, VAMP721 and VAMP722		Membrane fusion	PIN1 and PIN2		Root gravitropism	Gu et al., 2021; Zhang et al., 2021

### Endocytosis

**Figure 2:**
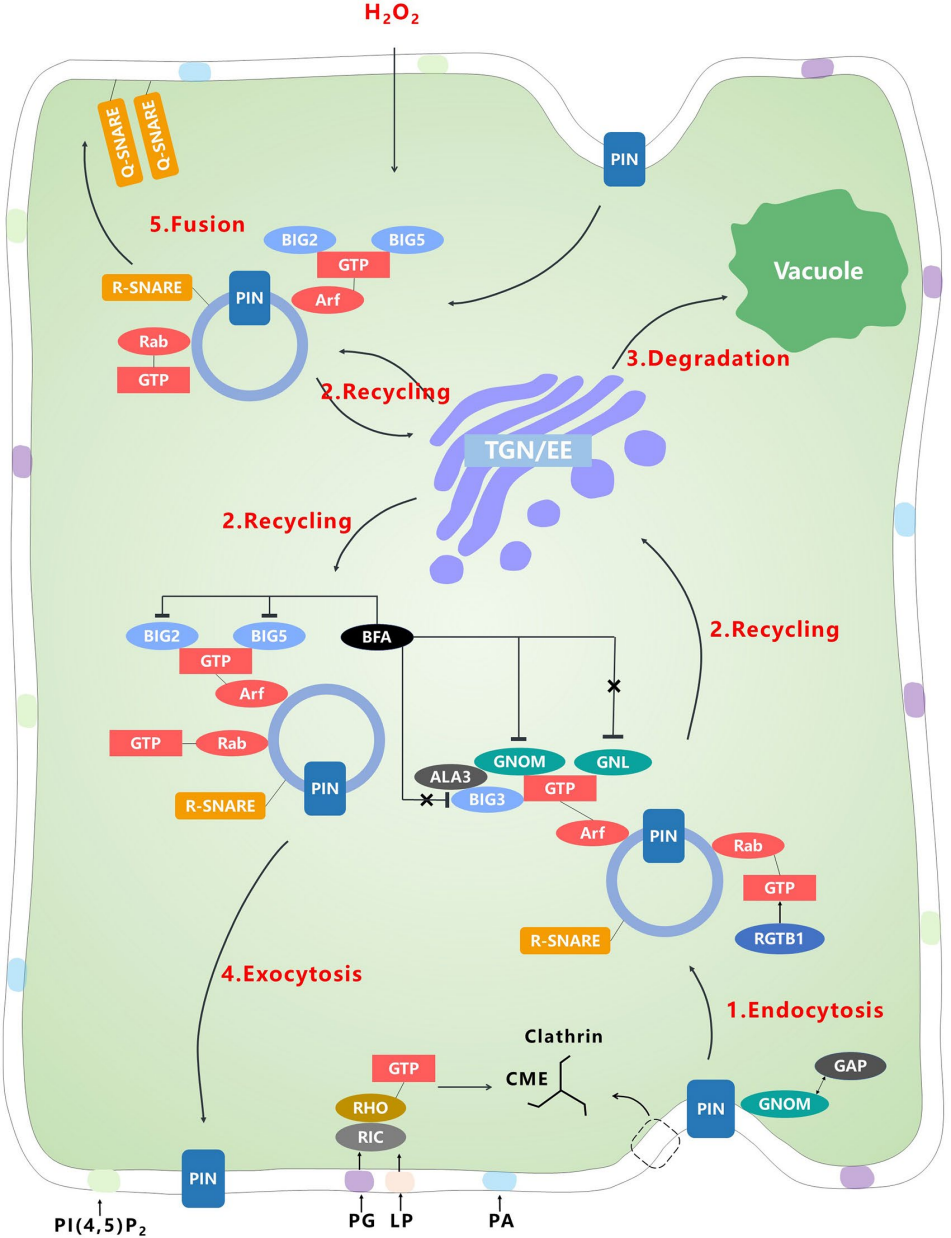
PINs can be recycled or degraded through endocytosis, subcellular trafficking and membrane fusion. In endocytosis, clathrin mediates the formation of vesicles. The PINs are then transported in the endosomal vesicles, in which many Arf and Rab proteins influence the destination of the PINs. These Arf and Rab proteins are further regulated by GEF and GAP, including GNOM, a PINs-specific AEF-GEF. Finally, the vesicles fuse to the destination membrane, regulated by SNARE. In addition, lipids are involved in the binding of these membrane-associated proteins. Notably, among these factors that may affect the polarity of PINs, only GNOM is primarily involved in the recycling basal PINs.

To recycle or degrade PINs, the first trafficking step is endocytosis (Figure 2). Clathrin-coated vesicles are carriers for membrane vesicular transport, in which the clathrin unit is a triskelion-like structure, comprising heavy and light chains [CLATHRIN HEAVY CHAIN (CHC) and CLATHRIN LIGHT CHAIN (CLC) proteins] with three arms that assist the clathrin to assemble into different size structures (Kirchhausen and Harrison, 1981; Ungewickell and Branton, 1981; Fotin et al., 2004). PIN recycling depends on clathrin-mediated endocytosis (CME). Clathrin heavy chain or light chain mutants may exhibit severe defects in PIN trafficking and polar localization, thereby affecting auxin distribution and auxin-related phenotypes, such as hook opening and hypocotyl phototropism (Kitakura et al., 2011; Yu et al., 2016; Zhang et al., 2017). Studies of the *clc2 clc3* double mutants show that disruption of clathrin formation influences hypocotyl growth by changing PIN3 lateral localization and causes PIN3 relocalization after exposure to blue light (Zhang et al., 2017; Hu et al., 2021). During CME, dynamin-related proteins, which assist in the vesicle isolation from the membrane, affect PIN polarity because they may be involved in CME of PINs from the cell plate (Mravec et al., 2011).

Lipids can also affect the CME process. The sterol-biosynthesis mutants*, cyclopropylsterol isomerase1-1 (cpi1-1)*, display defective PIN localization (Men et al., 2008). An additional type of signaling phospholipid, phosphatidylinositol (4,5)-bisphosphate [PI(4,5)P2], also influences PIN localization. PI(4,5)P2 mostly localizes to the PM and regulates many subcellular events. PI(4,5)P2 is mainly derived from phosphatidylinositol-4-phosphate (PI4P), which is synthesized by 11 phosphatidylinositol 4-phosphate 5-kinases (PI4P5K; Mueller-Roeber and Pical, 2002). The *pip5k1 pip5k2* double mutant exhibits a disruption of PIN recycling. This indicates that PI(4,5)P2, which influences the formation of clathrin vesicles, is required for the establishment of PIN polarity (Ischebeck et al., 2013). Besides, PIP5Ks are recruited by BREVIS RADIX (BRX), a plant-specific PM-localized protein, and a type of kinase that is PROTEIN KINASE ASSOCIATED WITH BRX (PAX), to influence the abundance of PINs at PM (Marhava et al., 2020). This interaction establishes the link between CME and kinases, and suggests that PI(4,5)P2 abundance may be the signal at the PM. In addition, lipids can affect other membrane proteins to influence PIN polarity. The endocytosis of PIN1 is regulated by the clustering of RHO-LIKE GTPASE (ROP6), which is influenced by lipid phosphatidylserine (LP), phosphatidylglycerol (PG) and the sterol-dependent clustering of TRANSMEMBRANE RECEPTOR KINASE 1 (TMK1), and ROP-INTERACTIVE CRIB MOTIF-CONTAINING PROTEIN 1 (RIC1; Chen et al., 2012; Han et al., 2018; Platre et al., 2019; Pan et al., 2020). Polyacidic phospholipids might impact on the binding between the PM and a 14–3-3 regulated protein, NON-PHOTOTROPIC HYPOCOTYL 3 (NPH3), to regulate the PIN polarity during phototropic growth of the hypocotyl (Keicher et al., 2017; Reuter et al., 2021).

### Vesicular transport

After endocytosis and during recycling, PINs are located in the vesicles, where many proteins may influence the trafficking of PINs (Figure 2). The cytoskeleton and molecular machinery control the movement of vesicles, but it is the small GTPases, Arf and Rab proteins that regulate intracellular transport by connecting membranes to the cytoskeleton machinery and labeling vesicles for their final destination (Khan and Menetrey, 2013; Kjos et al., 2018). In addition, although Arf and Rab mainly regulate intracellular trafficking, they can also influence other GTPase, such as Rho, which usually regulates the actin cytoskeleton and may influence the localization of PIN proteins (Han et al., 2018; Kjos et al., 2018).

Arf binds to the vesicular membrane weakly in the GDP form and binds tightly in the GTP form. After Arf binds to the membrane, the GDP/GTP EXCHANGE FACTOR FOR SMALL G PROTEINS OF THE ARF CLASS (ARF-GEFs) are recruited to change the Arf type from GDP to GTP (Donaldson and Jackson, 2000). Brefeldin A (BFA) blocks the guanine-nucleotide exchange reaction to inhibit vesicle trafficking reversibly, ARF-GEFs in *A. thaliana* were initially classified as BFA-INHIBITED GEFs (BIG) and Golgi BFA resistance factor (GBF) initially (Donaldson and Jackson, 2000). Among GBFs, GNOM is BFA sensitive (Naramoto et al., 2010). GNOM is mostly localized to the Golgi apparatus and partly to the PM and TGN/EE (Naramoto et al., 2010, 2014). GNOM regulates the endocytosis and recycling of PIN1, which further affects PIN1 polarity (Steinmann et al., 1999; Geldner et al., 2003). Inhibition of GNOM by BFA leads to apical localization of PIN1, but the engineered BFA-insensitive GNOM only causes the recycling of PIN1 to become BFA insensitive, not that of other proteins (Geldner et al., 2003). Localization of PIN2 is not completely determined by GNOM. It requires additional homologs, such as GNOM-LIKE1 (GNL1), which also is localized to the Golgi apparatus but differs from GNOM (Richter et al., 2007; Teh and Moore, 2007; Kleine-Vehn et al., 2008). GNL1 plays an important and conserved role in ER-Golgi trafficking (Richter et al., 2007; Teh and Moore, 2007). In contrast, whereas GNOM is functionally redundant in ER-Golgi trafficking, the primary role of GNOM is the recycling of basal PINs (in root cells; Richter et al., 2007; Teh and Moore, 2007). There is evidence that GNOM and GNL1 are involved in the early secretory of PIN1 in the root, which contributes to the basal localization of PIN1 (Luschnig and Vert, 2014; Doyle et al., 2015). In addition, other factors acting in GNOM-independent endosomes can regulate PIN polarity. For example, recycling of PIN2 requires SORTING NEXIN 1 (SNX1), CLASP, and VACUOLAR PROTEIN SORTING 29 (VPS29), a factor downstream of GNOM (Jaillais et al., 2006, 2007; Ambrose et al., 2013). Some research indicates that the PM-localized GNOM and VAN3, an ARF-GTPase-activating protein (GAPs), which counteracts GEF, are also required for endocytosis (Naramoto et al., 2010; Naramoto and Kyozuka, 2018).

In the BIG class, BEN1/BIG5 and BEN3/BIG2 mainly co-localize in the TGN/EE, and influence the early trafficking and polar localization of PIN1 (Richter et al., 2014; Jonsson et al., 2017; Kitakura et al., 2017; Matsuura et al., 2020; Zhang et al., 2020). BEN1/BIG5 is involved in hydrogen peroxide-induced relocalization of PIN2 (Zwiewka et al., 2019). In addition, ALA3, a phospholipid flippase, produces and maintains the asymmetric distribution of phospholipids. The ALA3 protein directly interacts with GNOM and BIG3, and affects the transport and polarity of PINs (Best et al., 2019; Zhang et al., 2020). Similar to Arf, Rab-related pathways also involve RAB-GEF and RAB-GAP to activate or inactivate Rab (Martiniere and Moreau, 2020). Although no RAB-GEF is known to regulate polar trafficking of PINs, some evidence indicates that the Rab pathway is involved in vesicle circulation and degradation of PINs, and the change in Rab pathway affects the polar growth of root hair cells (Preuss et al., 2006; Feraru et al., 2012; Ivanov et al., 2014; Rodriguez-Furlan et al., 2019). Knocking out *RGTB1*, which catalyzes the Rab prenylation to assist Rab to bind more stably to the membrane, impairs the recycling of PIN1 and PIN3 (Rojek et al., 2021a,b).

Most of the factors that affect the polar trafficking of PINs are localized in the TGN. Recently, FORKED1 (FKD1), FORKED1-LIKE (FL), and SCARFACE (SFC) were detected in the TGN and were proposed to influence the secretory pathway that transports PIN1 to the apical PM during leaf vein development (Mariyamma et al., 2018). CHOLINE TRANSPORTER-LIKE 1 (CTL1) partially localizes to the TGN and can mediate choline transport to impact the homeostasis of membrane lipids. Interestingly, CTL1 regulates trafficking of PIN1 and PIN3 by acting on both secretory vesicles and clathrin-coated vesicles in the TGN (Wang et al., 2017). In addition, sphingolipids mediate polar sorting of PIN2 at the TGN by changing the level of PI4P, and thus the lipid can also affect PIN2 recycling (Ito et al., 2021).

### Membrane fusion

Fusion of the vesicles to the destination membrane is the final step in the vacuolar protein transfer. The PINs then may be degraded, recycled or remained in the membrane to form or change the polarity (Figure 2). Before fusion, the exocyst complex is responsible for the initial attachment of the vesicle to the PM (Saeed et al., 2019). Studies of *sec6, sec8,* and *exo70* mutants, show that the exocyst directly influences polar exocytosis of PINs (Drdova et al., 2013; Tan et al., 2016). Membrane fusion is then mediated by SOLUBLE N-ETHYLMALEIMIDE-SENSITIVE FUSION (NSF) PROTEIN ATTACHMENT PROTEIN RECEPTOR (SNARE). SNARE proteins can be classified as Q-SNARE and R-SNARE. Q-SNARE proteins are normally localized to the target membrane and R-SNARE proteins are in the TGN/EE (Fasshauer et al., 1998). When the three Q-SNARE and one R-SNARE proteins bind together, the membrane vesicles are fused (Jahn et al., 2003; Pratelli et al., 2004). The R-SNARE triple mutants, *vamp714 vamp721 vamp722*, shows defective polarity of PIN1 and PIN2 (Gu et al., 2021; Zhang et al., 2021). Notably, the polarity of PIN1 and PIN1-mediated polar auxin transport also requires AtNSF, which regulates leaf serration (Tang et al., 2021). It is generally accepted that SNAREs are involved in the Rab GTPase pathway, by interacting with Rab proteins to enable the fusion of membrane vesicles (Ebine and Ueda, 2009; Ohya et al., 2009; Ebine et al., 2011). No data are available on the interaction between ARF-GEF and SNAREs, but ARF-GAP is capable of interacting with SNAREs (Rein et al., 2002).

Overall, the function of each subcellular-trafficking related factor has not been fully explored. These factors can influence more than one process: clathrin can influence the recycling; GNOM and VAN3 are also involved in endocytosis (Naramoto et al., 2010; Robinson and Pimpl, 2014). Thus, although an intracellular trafficking model to explain the localization of PINs is accepted, it is not a complete theory. Many additional factors can influence the polarity of PINs and the relationships among the factors that already identified are not clearly demonstrated. Therefore, large scale exploration of the interactions or networks of these proteins is required (Tang et al., 2020). In addition, since vesicular trafficking is conserved in many organisms, the focus should be not only on PINs, or plants, but also on the regulatory mechanisms documented in other organisms (Glanc et al., 2021).

## Post-translational modification of PINs

### Phosphorylation and dephosphorylation

**Figure 3:**
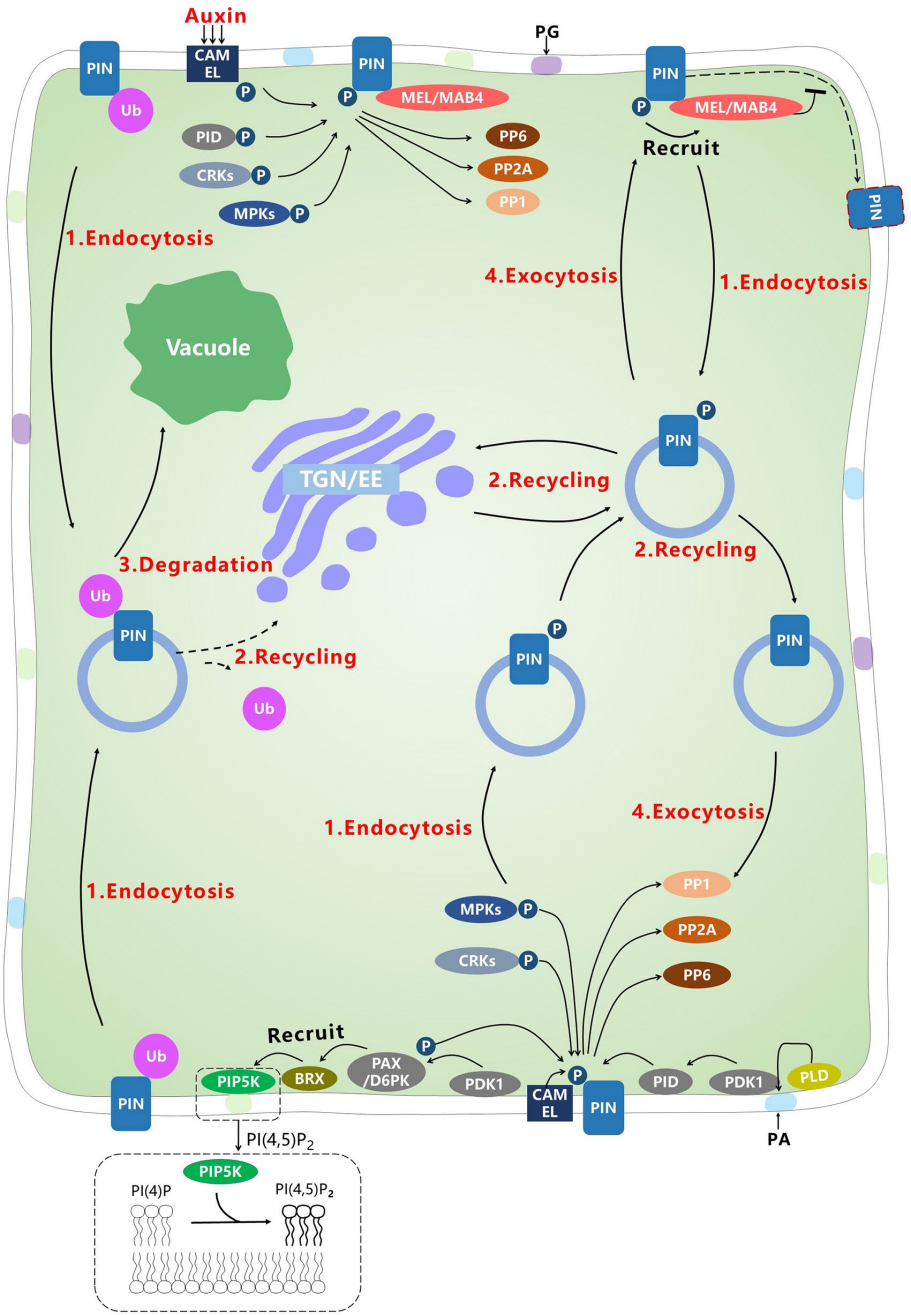
Post-translational modification regulates the localization of PINs. Kinases such as PID, D6PK, MPKs, and CRKs, directly phosphorylate PINs to change the polarity and transport activity of PINs. PAX recruits BRX to regulate the formation of PI(4,5)P2, which further influences the endocytosis of PINs. Phosphorylated PINs can recruit MAB4/MEL to maintain or change the localization of PINs through BFA-sensitive or BFA-insensitive endosomal vesicles. In addition, dephosphorylation regulated by phosphatases acts antagonistically to the changes caused by kinases. Among these proteins, only PAX/D6PK is localized only found to the basal PM.

The activity and localization of long PIN proteins can be regulated through phosphorylation by at least three different types of protein kinases (Table 4): SERINE/THREONINE-PROTEIN WITH HOMOLOGY TO MAMMALIAN PROTEIN KINASE A, CGMP-DEPENDENT KINASE, AND PROTEIN KINASE C (AGC kinases), MITOGENACTIVATED PROTEIN (MAP) KINASES (MPKs), and Ca^2+^/CALMODULIN-DEPENDENT PROTEIN KINASE-RELATED KINASES (CRKs). In addition, certain phosphatases dephosphorylate PINs (Figure 3).

**Table 4:** Kinases that may affect PIN polarity.

Kinase	Distribution	Influence on PINs localization		Influence on PINs transports activity	Phosphorylation sites	References
		Loss of function	Overexpression			
PID, WAG1, WAG2	None-polarity	Apical-to-basal localization	Basal-to-apical localization	Activate	S231, S252 and S290 (vitro and vivo) in PINs	Benkova et al., 2003; Friml et al., 2004; Kleine-Vehn et al., 2009; Dhonukshe et al., 2010; Huang et al., 2010; Zourelidou et al., 2014
D6PKs	Basal membrane (root cell)	Unchanged	Unchanged	Activate	S231, S252, S290, S215 and S271, mainly S215 and S271 (*in vitro* and *in vivo*) in PINs	Zourelidou et al., 2009; Barbosa et al., 2014; Zourelidou et al., 2014
PAX	Basal membrane (root cell)	Unchanged		Activate	In PINs	Marhava et al., 2018
PDK1	Basal membrane (root cell)	Unchanged		Activate	In PID, D6PKs andPAX	Zegzouti et al., 2006; Rodriguez et al., 2010; Xiao and Offringa, 2020
MPK6	None-polarity	Apical-to-basal localization			S337, T227, T248 and T286 in PINs (*in vitro* and *in vivo*)	Jia et al., 2016; Dory et al., 2018
MPKK7	None-polarity		Reduce basal localization		In MPK6 and other MPKs	Jia et al., 2016
CRK5	None-polarity	Only change PIN2 localization in root transition region			S252 / S253 of PIN1, S271 of PIN4, and S431 and S277/S278 in PIN7 (supposed)	Rigo et al., 2013; Baba et al., 2019a,b
CAMEL, CANAR		Change PIN1 localization			T129, T234, S240, T257, and S408 in PINs	Hajny et al., 2020

### AGC kinases

PINOID (PID), a member of the AGC family, was first found to be related to PINs. In *A. thaliana*, there are four members of the PID family (PID, PID2, WAVY ROOT GROWTH 1 (WAG1) and WAG2; Galvan-Ampudia and Offringa, 2007). Given that the *pid* mutants are phenotypically similar to *pin* mutants, the relationship between these two genes was studied (Bennett et al., 1995). *PID* was first cloned and implicated as a negative regulator in auxin expression signaling (Christensen et al., 2000). Benjamins et al. (2001) used overexpression lines to demonstrate that PID was not associated with the expression signaling, but may be a positive regulator of auxin efflux carriers (Benjamins et al., 2001). Overexpression of PID can over-phosphorylate the three conserved serine sites of PINs, S1, S2, and S3 (S231, 252, and 290 in PIN1), thereby altering the localization of PIN proteins from the basal to apical PM; in addition, the PINs showed apical-to-basal localization in *pid* mutants (Friml et al., 2004; Huang et al., 2010). Given that the distribution of PID is non-polar but that of PIN is polar, Dhonukshe et al. proposed a model in which PINs are not first polarly distributed at first, and thereafter reach the apical side when phosphorylated by PID. However, this model has not been clearly demonstrated (Dhonukshe et al., 2010). In addition, WAG1 and WAG2 are functionally redundant to PID (Dhonukshe et al., 2010). WAG1 and WAG2 phosphorylate the same region as PID does and show similar localization to PID (Dhonukshe et al., 2010). These three kinases have similar functions in root development, namely apical hook opening and photoresponse (Dhonukshe et al., 2010; Willige et al., 2012; Haga et al., 2014). More importantly, these three kinases all show BFA-insensitive localization and phosphorylate PINs to change their localization through BFA-insensitive processes (Dhonukshe et al., 2010). PID2 has not been well studied, but according to evolutionary reconstruction and the observation that photoreactions in *pid pid2 wag1 wag2* quadruple mutants are significantly more impaired than *pid wag1 wag2* triple mutants, the function of PID2 may be similar to that of the other three kinases (Tang et al., 2020). Despite the shift in distribution, PID-related phosphorylation can stimulate the activity of PINs transport (Zourelidou et al., 2014). Some studies have reported that phosphorylated PIN1 is detectable at the basal PM in the wild-type and *pid* mutants, suggesting that PID does not alter PIN polarity but only activates PIN transport activity (Weller et al., 2017). The change in polarity might result from other factors that interact with PID in PID-overexpression lines (Haga et al., 2014). Thus, although genetic and biochemistry experiments have provided substantial information on PID-related phosphorylation, the mechanism of the change in PIN polarity remains unknown.

Two important factors upstream and downstream of PID that regulate the PIN polarity should be mentioned. The first protein is associated with lipids. PHOSPHOLIPASE D (PLD) is responsive to many environmental signals. Under salt stress, PLD ζ 2 is involved in the endocytosis of PIN2 (Li and Xue, 2007; Galvan-Ampudia et al., 2013). Also, salt stress activates PLD α 1 and PLD Δ, which will generate phosphatidic acid (PA). The PA then binds to PID to phosphorylate PIN2, and increase the activity of PIN2 (Wang et al., 2019). A recent study observed that MACCHI-BOU4 / MAB4(ENP1)-LIKE (MAB4/MEL), to which belongs to the MAB4 protein family and was originally considered to be associated with PIN-related auxin transport, is recruited by phosphorylated PINs, which in turn form a positive feedback loop, thus promoting continuous phosphorylation of PINs by the AGC3 family to confine PIN to polar regions (Glanc et al., 2021).

The D6 PROTEIN KINASE (D6PK) belongs to the AGC family. It is generally accepted that D6PK as well as D6PKL1, D6PKL2, and D6PKL3 phosphorylate S1, S2, S3, S4, and S5 of PINs (S271, D215 in PIN1/S215 in PIN3; Zourelidou et al., 2014). PID tends to phosphorylate S1, S2, and S3 first, whereas D6PK preferentially phosphorylates S4 and S5 (Zourelidou et al., 2009, 2014). D6PK also phosphorylates S1, S2, and S3, indicating that the function of these two kinases is redundant (Zourelidou et al., 2014). Surprisingly, S4 and S5 are not conserved in PIN2, and S5 may be replaced by a site that is naturally phosphorylated in PIN1 (Zourelidou et al., 2014). Although *d6pk* mutants show some similar morphological phenotypes to *pid* mutants, some differences between D6PK and PID have been noted. Loss-of-function and overexpression of D6PK do not change PIN polarity (Zourelidou et al., 2009). In addition, unlike PID, D6PK is localized only in the basal PM (in root cells) and only phosphorylates the basal PINs (Barbosa et al., 2014). These phosphorylated PIN proteins as well as D6PK are sensitive to BFA treatment, but D6PK may be more closely associated with GNOM-dependent recycling than PINs (Weller et al., 2017). An additional D6PK/D6PKL-related kinase is PAX. BRX is recruited by PAX to the basal PM, where BRX normally binds to PAX and impedes PIN transport at low auxin concentrations (Marhava et al., 2018; Xiao and Offringa, 2020). When the BRX abundance declines as auxin concentration increases, PAX phosphorylates PINs to promote auxin transport (Xiao and Offringa, 2020). 3-PHOSPHOINOSITIDE-DEPENDENT PROTEIN KINASE 1 (PDK1), may provide the link between PID and D6PK. PDK1 is also a member of the AGC family, and often acts as an upstream signal to phosphorylate and activate PID, D6PK, and PAX to modulate the polarity of PINs (Zegzouti et al., 2006; Tan et al., 2020).

### MPKs

MAPK/MPKs are a conserved signaling kinase family that regulates many processes in plants, such as development and response to diverse environmental stresses (Rodriguez et al., 2010; Lin et al., 2021). This family always acts as a signal cascade. For example, MPK is phosphorylated by MPK KINASE (MPKK), which is further phosphorylated by MPKK kinase. MAP KINASE KINASE 7 (MKK7) is an upstream kinase of MAP6, and MKK7–MAP6 phosphorylates an unconservative site of PIN1, S337, to affect basal PM localization of PIN1 in the process of branch development (Jia et al., 2016). Interestingly, three conserved sites, T227, T248, and T286 of PIN1, which are located near the PID phosphorylation sites (S1–S3) and are components of the TPRXS motifs at S1–S3, are phosphorylated by MAP6 to influence the recycling of PIN1 (Dory et al., 2018). This finding indicates that PID may be associated with MPKs. However, the PM localization of PIN1 is abolished in most cells of MKK7 overexpression transformants, which differs from PID-and D6PK-overexpression transformants (Dory et al., 2018). Thus, MPKs may regulate PIN in a manner different from PID.

### CRKs

Calcium ions are an important second messenger involved in gravitropism (Perera et al., 2006). CRKs are kinases responsive to Ca^2+^. CRK5 is localized to the PM and phosphorylates PINs to regulate plant development (Rigo et al., 2013). In the root transition region of *crk5-1* mutants, the amount of PIN2 is reduced in the upper PM of epidermal cells, and is increased in the apical PM in the cortex, which is similar to the response in the wild type treated with a low concentration of BFA, although the localization of PIN1, 3, 4, and 7 is unchanged (Rigo et al., 2013). In addition, CRK5 may contribute to other mechanisms of PIN phosphorylation PINs. CRK5 regulates hypocotyl hook development, possibly through phosphorylation of PIN3, and embryo development through phosphorylation of PIN1, 4, and 7 (Baba et al., 2019a,b). No *in vivo* or *in vitro* evidence for the phosphorylation sites is available, although CRK5 can phosphorylate PIN1, 2, 3, 4, and 7 *in vitro* (Baba et al., 2019b). CRK5 is only supposed to phosphorylate S252 or S253 of PIN1, S271 of PIN4 and S431 and S277/S278 of PIN7 (Baba et al., 2019b). In addition to CRK5, CPK29 phosphorylates most PM-localized PINs to regulate the PIN polarity through BFA-insensitive recycling (Lee et al., 2021).

### Other kinases

Other types of kinases affect PIN polarity, too. CANALIZATION RELATED AUXIN-REGULATED MALECTIN-TYPE RLK (CAMEL) and CANALIZATION-RELATED RECEPTOR-LIKE KINASE (CANAR) are kinases that influence PIN1 polarity and respond to auxin (Hajny et al., 2020). The kinase may phosphorylate T129, T234, S240, T257, and S408 of PIN1, which is different from other kinases (Hajny et al., 2020). The loss-of-function of CAMEL or CANAR causes defective PIN1 polarity (Hajny et al., 2020). However, compared with other types of kinases, this receptor-like kinase is poorly studied. This represents a novel research focus to gain insight into the relationships among kinases.

### Phosphatases

The function of phosphatases is to dephosphorylate proteins and contribute to the homeostasis of reversible phosphorylation. PROTEIN PHOSPHATASE 2A (PP2A) is a heterotrimeric protein consisting of two regulatory subunits, A and B. It is involved in many processes that counteract PID. The PP2A A subunit is able to dephosphorylate PIN1 to change the polarity. Overexpression of PID further impairs mutants of *pp2a* subunits, whereas knocking out PID rescues *pp2a* A subunit mutants (Michniewicz et al., 2007b). In addition, *pp2a* A subunit mutants show basal-to-apical localization of PIN1, which is similar to the response to PID overexpression (Michniewicz et al., 2007b). Interestingly, other protein phosphatases, such as PP6 and PP1 also affect the PIN polarity. PHYTOCHROME-ASSOCIATED SER/THR PROTEIN PHOSPHATASE 1 (FyPP1) interacts with PP2A A subunit to form the PP6 heterotrimeric holoenzyme complex to regulate the phosphorylation of PIN proteins by antagonizing PID (Dai et al., 2012). An additional subunit of PP1, TYPE-ONE PROTEIN PHOSPHATASE 1 (TOPP1), acts antagonistically to PID (Guo et al., 2015). However, in contrast to kinases, few studies of phosphatases have been reported, and thus, the network existing among these phosphatases remains unknown.

To date, GNOM is the only intracellular trafficking-related protein known that specifically regulates PIN recycling. However, none of the kinases that can regulate the PIN polarity is recycled through GNOM-dependent trafficking. Only D6PK is associated with GNOM, but it does not change the PIN polarity. PID and GNOM have an antagonistic effect on the localization of PINs. PID can phosphorylate apical PINs and maintain the phosphorylation abundance to decrease GNOM-dependent subcellular trafficking of PINs (Kleine-Vehn et al., 2009). However, in response to BFA treatment, to which D6PK is sensitive but the PID is insensitive, PIN1 phosphorylation is not maintained in the wild type, but is maintained in engineered BFA-insensitive GNOM mutants, even though some of the PID proteins are localized to the basal PM and potentially may phosphorylate PINs (Barbosa et al., 2014). Thus, protein phosphatases may play a role in this process (Barbosa et al., 2014). Despite the Arf, Rab may also be associated with phosphorylation. Rab5 in endocytosis may be involved in phosphorylation (Weller et al., 2017). Notably, it is uncertain whether phosphorylation by each kinase can change the polarity of PIN proteins by intracellular trafficking directly from one part to another. This is because knocking out and overexpressing some kinases cause a clear change in PIN polarity (in *pid* mutants is basal and in PID-overexpression mutants is apical; in *crk5* mutants is apical), but the phosphorylated PINs are observed on each side of the PM (Weller et al., 2017). The phosphorylated PINs may be confined to one side, newly synthesized PINs are secreted *de novo* to this side, and the PINs in other sides are degraded. This is because phosphorylated PINs can recruit MAB4/MEL to form positive feedback to limit lateral diffusion and maintain phosphorylation, and PIN2 is not recycled from the basal to the apical PM, but is newly secreted to the apical PM after cell division (Weller et al., 2017; Glanc et al., 2018).

### Other post-translational modification

S-Nitrosylation (SNO), the addition of nitric oxide (NO), is controlled by NO concentrations and denitrosylation catalyzed by thioredoxin and S-NITROSOGLUTATHIONE REDUCTASE (GSNOR; Feechan et al., 2005; Sengupta and Holmgren, 2013). The lack of GSNOR1 inhibits the endocytosis of PINs by an uncertain mechanism. Both GNOM and PID have hypothetical SNO modifications (Ni et al., 2017; Sanchez-Vicente et al., 2021). The influence of GSNOR also indicates that PIN polarity may be affected by NO. In addition, ubiquitination of PINs influences the recycling and degradation of PINs. Loss of K63 ubiquitination of PIN2 interferes with its transport to the vacuole (Leitner et al., 2012).

## Conclusion

The PIN-mediated short-distance transport of auxin is important to regulate plants growth and for tropic responses. A model to explain the distribution of PINs has been formulated. PINs are secreted, recycled or degraded through intracellular trafficking, in which GNOM plays a role to recycle the basal PINs, and PINs can be phosphorylated to change the polarity. However, some gaps in this model remain. Only GNOM or GNOM-like ARF-GEFs are known to be responsible for the recycling, but additional factors, such as the myosin and other Arf and Rab proteins are still poorly studied (Abu-Abied et al., 2018). Given that previous research on GNOM has invariably used BFA to induce the changes, the use of other inhibitors, such as endosidin 4, are also worth exploring (Kania et al., 2018). In the phosphorylation and dephosphorylation section, the model lacks the decisive evidence to determine which role, the change in PIN polarity or in PIN transport activity, is dominant. Furthermore, many kinases or protein phosphatases are functionally redundant but somewhat different in their target sites. In addition, the relationship between phosphorylation and trafficking remains unknown. It is uncertain how the locations of PINs are changed, after phosphorylation. Also, there are many other relevant factors not incorporated in this model. For example, ubiquitin can regulate whether PINs are directed to the vacuoles for degradation, the distribution of lipids, such as PI4P, PI(4,5)P2 and PA, can affect the localization of kinases and PIN, and the auxin fluxing through plasmodesmata directly challenges the PIN transport (Deak et al., 1999; Leitner et al., 2012; Tejos et al., 2014; Stanislas et al., 2015; Barbosa et al., 2016; Gao et al., 2020; Mellor et al., 2020). Certain environmental signals have been reported to affect PINs. For instance, PLD responds to environmental change to influence kinases (Wang et al., 2019). Nevertheless, these pathways do not form a network. An additional important point is to consider components from other organisms. Studies on Glut4 (GLUCOSE TRANSPORTER 4) in mammalian cells, CDC42-dependent symmetry-breaking pathway in yeast, and the TRANSPORT PROTEIN PARTICLE (TRAPP) complex all show the importance of referring to the components from other organisms, because subcellular trafficking is conserved among organisms and some components of PIN trafficking machinery are evolutionary conserved (Garcia et al., 2020; Glanc et al., 2021).

## Author contributions

SC wrote the original manuscript and made the figures. SC and YW revised and completed the writing of the manuscript. All authors contributed to the article and approved the submitted version.

## Funding

This work was supported by Hainan Yazhou Bay Seed Laboratory (B21HJ0220), National Natural Science Foundation of China (31871537 and U2003115) and Zhejiang Provincial Natural Science Foundation of China (LR21C020001).

## Conflict of interest

The authors declare that the research was conducted in the absence of any commercial or financial relationships that could be construed as a potential conflict of interest.

## Publisher’s note

All claims expressed in this article are solely those of the authors and do not necessarily represent those of their affiliated organizations, or those of the publisher, the editors and the reviewers. Any product that may be evaluated in this article, or claim that may be made by its manufacturer, is not guaranteed or endorsed by the publisher.
